# Patterns of treatment and outcome of ductal carcinoma in situ in the Netherlands

**DOI:** 10.1007/s10549-020-06055-w

**Published:** 2021-01-01

**Authors:** Jacky D. Luiten, Ernest J. T. Luiten, Maurice J. C. van der Sangen, Willem Vreuls, Lucien E. M. Duijm, Vivianne C. G. Tjan-Heijnen, Adri C. Voogd

**Affiliations:** 1 Department of Surgery, Elisabeth-Tweesteden Hospital, Hilvarenbeekseweg 60, 5022 GC Tilburg, The Netherlands; 2 School for Oncology and Developmental Biology, Research Institute GROW, Maastricht University, PO Box 616, 6200 MD Maastricht, The Netherlands; 3 Department of Surgical Oncology, Amphia Hospital, Molengracht 21, 4818 CK Breda, The Netherlands; 4 Department of Radiotherapy, Catharina Hospital, Michelangelolaan 2, 5623 EJ Eindhoven, The Netherlands; 5 Department of Pathology, Canisius Wilhelmina Hospital, Weg door Jonkerbos 100, 6532 SZ Nijmegen, The Netherlands; 6 Department of Radiology, Canisius Wilhelmina Hospital, Weg door Jonkerbos 100, 6532 SZ Nijmegen, The Netherlands; 7 Dutch Expert Centre for Screening, Wijchenseweg 101, 6538 SW Nijmegen, The Netherlands; 8 Department of Internal Medicine, Division of Medical Oncology, Research Institute GROW, Maastricht University Medical Centre, PO Box 5800, 6202 AZ Maastricht, The Netherlands; 9 Faculty of Health Medicine and Life Sciences, Department of Epidemiology, Maastricht University, PO Box 616, 6200 MD Maastricht, The Netherlands; 10 Department of Research and Development, Netherlands Comprehensive Cancer Organization, Godebaldkwartier 419, 3511 DT Utrecht, The Netherlands

**Keywords:** Breast cancer screening, Ductal carcinoma in situ, Diagnostics, Treatment

## Abstract

**Purpose:**

To spare DCIS patients from overtreatment, treatment de-escalated over the years. This study evaluates the influence of these developments on the patterns of care in the treatment of DCIS with particular interest in the use of breast conserving surgery (BCS), radiotherapy following BCS and the use and type of axillary staging.

**Methods:**

In this large population-based cohort study all women, aged 50–74 years diagnosed with DCIS from January 1989 until January 2019, were analyzed per two-year cohort.

**Results:**

A total of 30,417 women were diagnosed with DCIS. The proportion of patients undergoing BCS increased from 47.7% in 1995–1996 to 72.7% in 2017–2018 (*p* < 0.001). Adjuvant radiotherapy following BCS increased from 28.9% (1995–1996) to 89.6% (2011–2012) and subsequently decreased to 74.9% (2017–2018; *p* < 0.001). Since its introduction, the use of sentinel lymph node biopsy (SLNB) increased to 63.1% in 2013–2014 and subsequently decreased to 52.8% in 2017–2018 (*p* < 0.001). Axillary surgery is already omitted in 55.8% of the patients undergoing BCS nowadays. The five-year invasive relapse-free survival (iRFS) for BCS with adjuvant radiotherapy in the period 1989–2010, was 98.7% [CI 98.4% – 99.0%], compared to 95.0% [CI 94.1% –95.8%] for BCS only (*p* < 0.001). In 2011–2018, this was 99.3% [CI 99.1% – 99.5%] and 98.8% [CI 98.2% – 99.4%] respectively (*p* = 0.01).

**Conclusions:**

This study shows a shift toward less extensive treatment. DCIS is increasingly treated with BCS and less often followed by additional radiotherapy. The absence of radiotherapy still results in excellent iRFS. Axillary surgery is increasingly omitted in DCIS patients.

## Introduction

Ductal carcinoma in situ (DCIS) is defined as an intraductal neoplastic proliferation of cells [1]. In most cases, DCIS of the breast are associated with the presence of suspicious calcifications on mammography. Calcifications are the result of precipitations of calcium salts in intraluminal secretions or necrosis of epithelial cells [2].

The nationwide biennial mammographic screening program in the Netherlands for women aged 50–70 years was set up between 1989 and 1996. In 1999, the upper age limit was extended to 75 years. The program led to a sharp increase in the detection rate of DCIS, which was reinforced by the replacement of screen-film mammography by full-field digital mammography in 2009–2010 [3, 4]. Autopsy studies have shown that DCIS often does not progress to invasive disease [5]. Sometimes a fraction of all preclinical DCIS may even regress spontaneously [6]. The aforementioned implies that part of the observed increase in the diagnosis and treatment of DCIS may be partly unnecessary and could be seen as overdiagnosis, thereby resulting in avoidable treatment-related morbidity [7, 8]. However, predicting which DCIS lesions will regress and which will proceed to invasive breast cancer is hardly possible yet. Therefore, almost all patients with DCIS undergo surgical treatment.

According to the guidelines, adequate treatment of DCIS consists of mastectomy or breast conserving surgery (BCS), pursuing complete microscopic tumor excision. In case of BCS additional whole-breast radiotherapy is standard of care [9, 10]. The recommendation for adjuvant radiotherapy is based on the results of several randomized controlled trials, showing a reduction of the incidence of both in situ and invasive local recurrence by half [11–13]. Fifteen-year ipsilateral local recurrence rates following BCS with adjuvant radiotherapy for DCIS vary between 7 and 11% [14]. Contralateral invasive breast cancer incidence fifteen years after DCIS diagnosis was approximately 6.5%, compared to 3.4% in the general population [14].

There is no evidence which supports performing axillary surgery in patients with pure DCIS in final pathology [15]. Axillary lymph node dissection (ALND), which used to be the gold standard, was therefore replaced by sentinel lymph node biopsy (SLNB) in the late 1990s. Today SLNB for patients with DCIS may be considered in the presence of clinical risk factors for an invasive component or for those who will undergo mastectomy [16].

The aim of this population-based study was to evaluate patterns of care in the treatment of DCIS in the Netherlands since the introduction of the national screening program with particular interest in the use of BCS, radiotherapy following BCS and the use and type of axillary staging. Additionally, we analyzed the risk of invasive local relapse in patients undergoing BCS.

## Methods

### Study population

In this population-based retrospective cohort study, data and records of all newly diagnosed women with DCIS in the Netherlands were retrieved from the Netherlands Cancer Registry (NCR). The NCR contains all new cases of in situ and invasive malignancies and data on patient, tumor and treatment characteristics [17]. Data are available on a national level since 1989. Patients were included in the NCR database, after notification by the nationwide Dutch Pathology Archive of Histo- and Cytopathology (PALGA) [18]. Specially trained data managers collected data from patient files in Dutch hospitals. The NCR routinely collected information on the occurrence of invasive relapse and the date of death. Follow-up for these endpoints was completed until January 2019.

In the Netherlands, the first round of a population-based screening program for breast cancer was implemented during 1989–1996, offering free-of-charge biennial mammography to women aged 50–70 years. Since 1999 women aged 70–75 years are also invited. Screen-film mammography was replaced by full-field digital mammography in 2009–2010. Since digital mammography a two-view mammography (medio-lateral-oblique view and cranio-caudal view) of each breast is obtained by a certified radiographer and the examination is assessed by two screening radiologists. For the current study, all screen-detected and clinically detected DCIS from January 1989 until January 2019 in women aged 50–75 years were included. Whether a patient was detected by screening was adequately registered since 2011.

For further analysis on subgroups we excluded all two-year cohorts with more than 20% missing data. This meant that for analyses of the type of local treatment and ALND patients treated before 1995 were excluded. For analysis on grade, we only included patients diagnosed from 2001 onwards. And for the analysis on SLNB, patients were included since 2005. Women with sentinel lymph node (SLN) involvement could not be included in our analyses, as in these cases the diagnosis of DCIS was overwritten by invasive breast cancer in the NCR database.

### Statistical analysis

Patients were categorized by two-year cohorts based on date of diagnosis.

Trends in breast surgery were studied and expressed as proportion of all patients per two-year cohort. The trends in use of adjuvant radiotherapy were expressed as proportions of all patients undergoing BCS per two-year cohort. Trends in use of radiotherapy was also categorized by grade. Trends in axillary surgery were categorized per type of axillary treatment, within those categories, trends in treatment were expressed as proportion of all patients. Trends in axillary treatment was also categorized by type of breast surgery. When trends were compared, missing data were excluded for all subgroups.

Statistical analyses were performed using SPSS, version 24.0 (SPSS, Inc., Chicago, USA). Chi-square analyses were performed to compare proportional differences in categorical variables between groups. *P*-values less than 0.05 were considered statistically significant. Kaplan–Meier analyses, were performed to estimate the cumulative risk of invasive local recurrence, expressed as invasive relapse-free survival (iRFS) [with 95% confidence interval] following BCS. Differences in the iRFS between periods of diagnosis and between patients with and without breast radiotherapy following BCS were compared by means of the two-tailed log-rank test.

## Results

Between January 1989 and January 2019, 30,417 women aged 50–74 years were diagnosed with DCIS in the Netherlands. Baseline characteristics of these patients are shown in Table 1. Since 2011, 75.1% (10,444/13,913) of all patients had been detected by the national screening program. The number of new cases increased from 379 in 1989–1990 to 3573 in 2017–2018. Of all patients 48.7% (11,238/23,065) was high grade, 34.5% (7953/23,065) intermediate grade and 16.8% (3874/23,065) low grade.

**Table 1:** Baseline characteristics

	Total (%)		Missing data excluded (%)
Period of diagnosis	*n* = 30,417		
1989–1990	379	(1,3)	
1991–1992	713	(2,5)	
1993–1994	968	(3,4)	
1995–1996	1.084	(3,8)	
1997–1998	1.270	(4,5)	
1999–2000	1.502	(5,3)	
2001–2002	1.555	(5,5)	
2003–2004	1.750	(6,2)	
2005–2006	1.872	(6,6)	
2007–2008	2.180	(7,7)	
2009–2010	2.818	(9,9)	
2011–2012	3.238	(11,4)	
2013–2014	3.690	(13,0)	
2015–2016	3.825	(13,5)	
2017–2018	3.573	(12,6)	
Screen detected^a^	*n* = 14,326		
Yes	10,444	(72.9)	
No	3,469	(24.9)	
Unknown	413	(2.9)	
Treatment^b^	*n* = 28,357		*n* = 27,783
Mastectomy	9,790	(34.5)	(35.2)
BCS + radiotherapy	13,859	(48.9)	(49.9)
BCS only	3,760	(13.3)	(13.5)
No treatment	374	(1.3)	(1.3)
Unknown	574	(2.0)	
DCIS grade^c^	*n* = 24,501		*n* = 23,065
Low	3,874	(15.8)	(16.8)
Intermediate	7,953	(32.5)	(34.5)
High	11,238	(45.9)	(48.7)
Unknown	1,436	(5.9)	
Axillary treatment			
ALND^b^	*n* = 28,357		*n* = 27,920
Yes	872	(3.1)	(3.1)
No	27,048	(95.4)	(96.9)
Unknown	437	(1.5)	
SLNB^d^	*n* = 21,196		*n* = 19,971
Yes	11,340	(39.1)	(56.8)
No	8,631	(30.4)	(43.2)
Unknown	1,225	(4.3)	

^a^Data available since 2011

^b^Data available since 1995

^c^Data available since 2001

^d^Data available since 2005

### Breast surgery and radiotherapy for DCIS

**Fig. 1:**
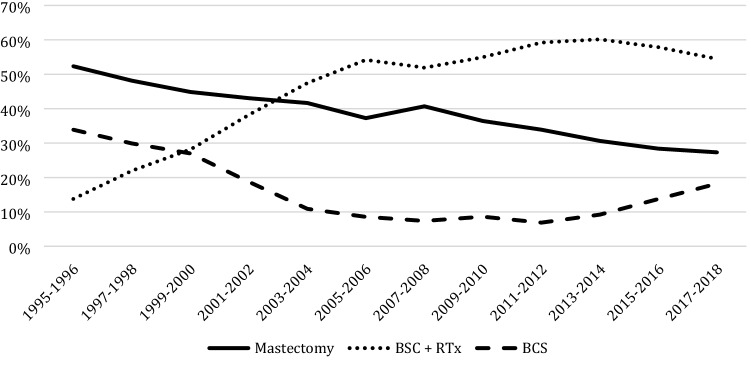
Type of surgical treatment. *BCS* breast conserving surgery, *RTx* radiotherapy

Since 1995 type of surgery was not specified in 2.0% (574/28,357) of all women diagnosed with DCIS, resulting in 27,783 women for whom type of treatment was registered. A mastectomy was performed in 9790 (35.2%; 9790/27,783) women, 17,619 (63.4%; 17,619/27,783) underwent BCS and 374 (1.3%; 374/27,783) did not receive any surgical treatment (Fig. 1). The percentage of patients undergoing BCS increased from 47.7% (460/965) in 1995–1996 to 72.7% (2474/3404) in 2017–2018 (*p* < 0.001).

**Fig. 2:**
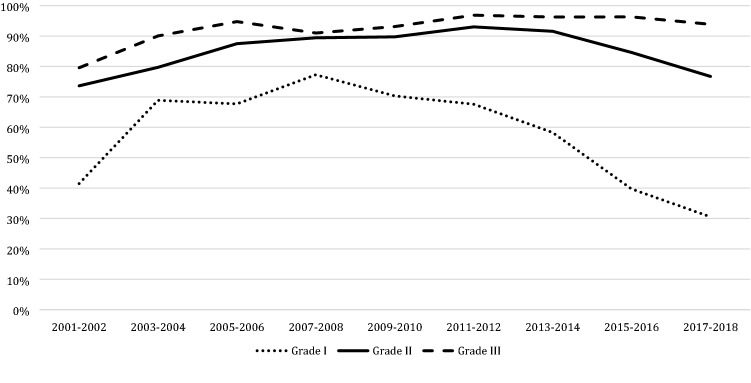
Trends in the use of radiotherapy after breast conserving surgery divide by grade since 2001

Among patients undergoing BCS, 78.7% (13,859/17,619) received adjuvant radiotherapy. This proportion increased from 28.9% (133/460) in 1995–1996 to 89.6% (1890/2110) in 2011–2012 (*p* < 0.001) and decreased again to 74.9% (1854/2474) in 2017–2018 (*p* < 0.001). Figure 2 shows the trend in use of radiotherapy after BCS since 2001, divided by DCIS grade. For low grade DCIS the number of patient receiving adjuvant radiotherapy increased from 41.5% (68/164) in 2001–2002 to 77.3% (170/220) in 2007–2008 (*p* < 0.001) which steadily decreased in more recent years to 30.5% (127/416) in 2017–2018 (*p* < 0.001). For intermediate and high grade DCIS, the use of radiotherapy remained rather stable (*p *= 0.72 and *p* = 0.09, respectively).

When comparing use of BCS in the different gradings in 2017–2018, 30.5% (127/416) of all low grade DCIS was treated with BCS including adjuvant radiotherapy, compared to 76.7% (758/988) of all intermediate grade DCIS (*p* < 0.001) and 93.9% (918/978) of all high grade DCIS (*p* = 0.002).

### Axillary surgery in DCIS

Since 1995, 872 (3.1%; 872/28,357) women underwent ALND. When excluding all missing data since 1995 (*n* = 437; 1.5%) the proportion of women undergoing ALND decreased over the years from 23.0% (223/1084) in 1995–1996 to less than 1% (27/2806) in 2009–2010 and later (*p* < 0.001). SLNB was performed in 11,340 women since 2005 (53.5%; 11,340/21,196). The proportion of patients undergoing SLNB rapidly increased since its introduction in 1997–1998 to 63.1% (2328/3690) in 2013–2014 and subsequently decreased to 52.8% (1888/3573) in 2017–2018 (*p* < 0.001).

**Fig. 3:**
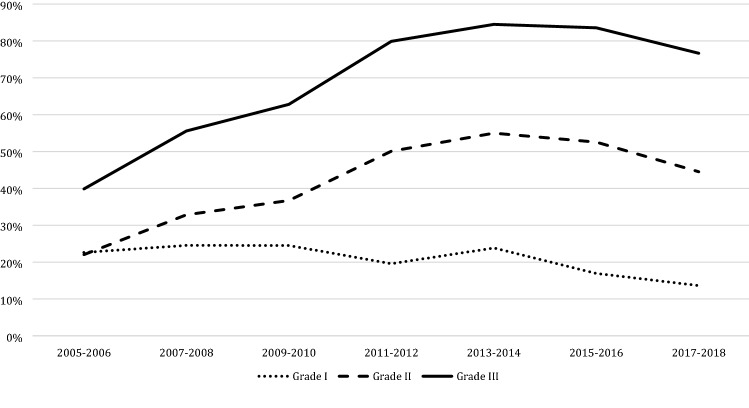
Trends in sentinel lymph node biopsy divided by grade

**Fig. 4:**
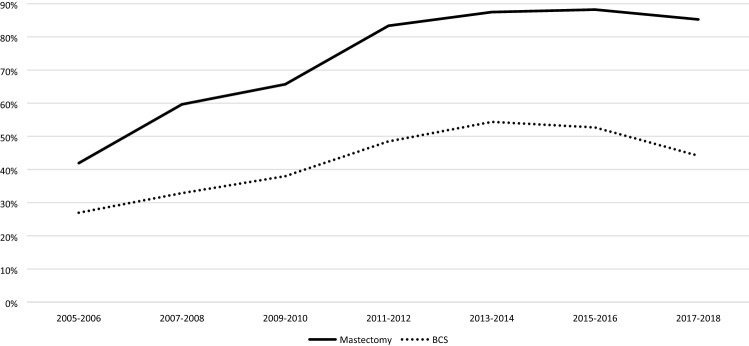
Trends in sentinel lymph node biopsy divided by type of surgical treatment

Overall, 20.2% (684/3393) of all patients with low grade DCIS underwent SLNB compared to 45.1% (3197/7087) of those with intermediate DCIS and 71.9% (7031/9778) of those with high grade DCIS (*p* < 0.001). Figure 3 shows the trend in SLNB use categorized by DCIS grade since 2005. When categorized by type of breast surgery, as shown in Fig. 4, 75.1% (5058/6732) of all patients who underwent a mastectomy since 2005 underwent SLNB, compared to 45.0% (6270/13,922) of the patients who underwent BCS (*p* < 0.001).

**Fig. 5:**
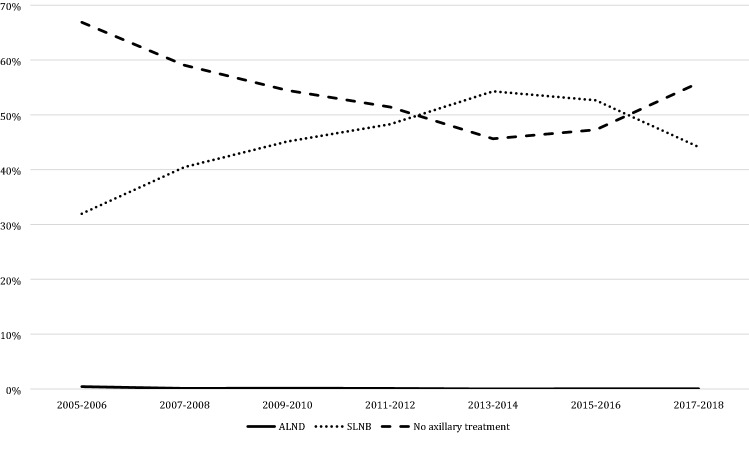
Trends in axillary treatment in breast conserving surgery. *ALND* axillary lymph node dissection, *SLNB* sentinel lymph node biopsy

In recent years, an increasing number of patients did not receive any axillary surgery. When focusing on BCS only, 52.3% (6.900 /13.187) of all women did not receive axillary surgery since 2005. In 2013–2014 axillary surgery was omitted in 45.6% (1140/2498), compared to 55.8% in 2017–2018 (1381/2474; *p* < 0.001; Fig. 5).

### Risk of invasive local recurrence

**Fig. 6:**
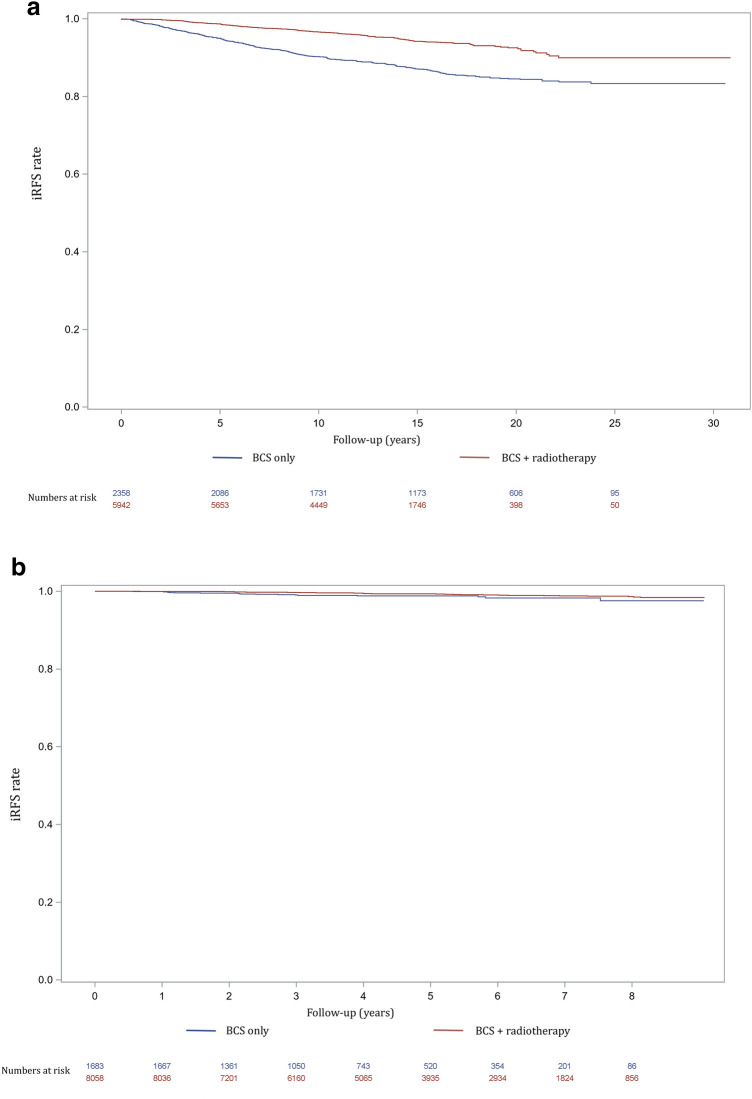
**a** Invasive relapse-free survival for the period 1989–2010, including numbers at risk, **b** Invasive relapse-free survival for the period 2011–2018, including numbers at risk

For the period 1989–2010, invasive relape-free survival (iRFS) analysis comparing BCS with and without adjuvant radiotherapy showed a five-year iRFS rate of 98.7% [CI 98.4% – 99.0%] (77 local recurrences) for women undergoing BCS with adjuvant radiotherapy, compared to 95.0% [CI 94.1% – 95.8%] (115 local recurrences) for women undergoing BCS only (*p* < 0.001; Fig. 6a). The ten-year iRFS rates were 96.6% [CI 96.2% – 97.0%] (192 local recurrences) vs. 90.2% [CI 89.0% – 91.5%] (215 local recurrences) respectively (*p* < 0.001). The fifteen-year iRFS rate was 94.2% [93.6%–94.8%] (261 local recurrences) compared to 87.1% [CI 85.6% – 88.6%] (265 local recurrences) respectively (*p* < 0.001).

For the period 2011–2018, the five-year iRFS rate was 99.3% [CI 99.1% – 99.5%] for women undergoing BCS with adjuvant radiotherapy, compared to 98.8% [CI 98.2% – 99.4%] for women undergoing BCS only (*p* = 0.01; Fig. 6b).

## Discussion

This large population-based study among patients aged 50–75 years shows a tenfold increase in the numbers of patients with DCIS in the Netherlands the last three-decades. The use of BCS increased from 47.7% in 1995–1996 to 72.7% in 2017–2018. Among patients undergoing BCS a sharp rise in the use of adjuvant radiotherapy was observed from 28.9% in 1995–1996 to almost 90% in 2011–2012, followed by a drop to 74.9% in the most recent years. The use of ALND decreased over the years while SLNB was introduced, which itself is increasingly replaced by no axillary surgery (55.8%) in patients undergoing BCS in 2017–2018.

Patients diagnosed with DCIS have become significantly more likely to receive BCS, which is in accordance with the findings in previously published studies [19, 20]. BCS has become the preferred surgical treatment for invasive breast cancer, after several randomized clinical trials had shown that BCS with radiotherapy offers the same overall survival rate as mastectomy alone [21, 22]. These findings may have had a positive effect on the use of BCS for DCIS as well. Another likely explanation might be that digital mammography screening detects lesions with smaller tumor sizes, which therefore may be more suitable for BCS [23, 24]. Unfortunately, our data did not include information on the extent of DCIS lesions.

Even though adjuvant radiotherapy following BCS is still considered as a standard therapeutic option in most treatment guidelines, the guidelines also state that radiotherapy can be omitted in cases of DCIS < 10 mm, low- and intermediate grade and excised with adequate margins [10]. We observed a decrease in the proportion of patients receiving additional radiotherapy the last five years. iRFS analyses showed that the risk of invasive local recurrence was dependent on the use adjuvant radiotherapy and that this association was much stronger for the patients diagnosed in the older period (1989–2010). The absolute difference in the 5 years risk of local recurrence between patients with and without adjuvant radiotherapy was only 0.5% (0.7% vs 1.2%, respectively) for the patients treated since 2011. A possible explanation for the low risk of invasive local recurrence in the recent period might be increased consensus on the relevance to obtain tumor-negative resection margins after BCS [25]. Another explanation might be the higher sensitivity of digital mammography, resulting in the detection of smaller lesions. This drives the discussion about omitting radiotherapy in a larger proportion of the patients undergoing BCS.

For the survival analysis 2011 was chosen as cut-off point, because digital mammography was fully implemented in the Netherlands since then.

Four randomized controlled trials on adjuvant radiotherapy in DCIS patients have been published [11, 12, 26, 27]. An overview of these trials showed that additional radiotherapy halves the risk of an ipsilateral breast event (invasive and non-invasive). However, it has not been shown to improve breast cancer overall survival [28].

If no survival benefit is found, the reduced risk of local recurrence following radiotherapy must be weighed against the disadvantages. The most common side effect of radiation is acute skin toxicity within weeks after treatment. Radiation can also have negative cosmetics effects due to development of skin pigmentation, telangiectasia, fibrosis and retraction [29]. Furthermore, breast radiotherapy might increase the risk of primary lung cancer among smokers and left-sided breast cancer radiotherapy is proven to be cardiotoxic [30, 31]. Because of this long-term side effect the average mean heart dose of left-sided whole-breast radiotherapy, which used to be 5.4 Gy [32], is much lower nowadays with the use of deep inspiration breath hold technique (reduction of 3.4 Gy) and partial breast irradiation [33].

Over the years, research focused on the identification of subgroups of patients with favorable features for whom the risk of invasive recurrence in the absence of radiotherapy is so low that radiotherapy can safely be omitted [34]. A recent observational study in 2016 suggests a possible survival benefit of radiotherapy, which may be most important when certain risk factors are present [35]. Factors such as tumor size, age and nuclear grade were used to produce a recurrence risk scoring system, known as the patient prognostic score. Significant improvements in survival after radiotherapy were only observed in patients with higher nuclear grade, younger age, and larger tumor size. The magnitude of the survival difference with radiotherapy was significantly correlated with this prognostic score (*p* < 0.001) [35]. Therefore, it is recommended to tailor radiotherapy on patient factors, tumor biology and the prognostic score [35, 36].

Since pure DCIS is not accompanied by nodal involvement, de-escalating axillary treatment in DCIS patients is justified. ALND is no longer part of the standard treatment for DCIS, as is also illustrated by our study, showing a replacement of ALND by SLNB. In most recent years we also observed a significant declined in axillary staging by any surgical procedure. The trend to omit SLNB is probably initiated by the rather low incidence of SLN involvement, ranging from 0 to 10% between different studies [37–39]. Unfortunately, we were not able to report on SLN involvement, as in case of any SLN involvement the diagnosis of DCIS was overwritten by invasive breast cancer in the NCR database.

Even if the SLN is found positive in patients with a preoperative diagnosis of DCIS, it is most frequently presents as isolated tumor cells or micrometastases (defined as small metastases sized 0.2–2.0 mm), which are of limited prognostic value on disease free and overall survival [15]. Therefore, we agree with a recent study by Van Roozendaal et al. who suggest to omit SLNB completely in patients with DCIS undergoing BCS, as preforming a delayed SLNB following lumpectomy if invasive cancer is shown is nowadays considered a feasible option [39]. In patients undergoing mastectomy, SLNB cannot reliably be performed afterwards and therefore may still need to be performed in DCIS patient undergoing a mastectomy [9].

Our study suggests that many clinicians use DCIS grade not only to consider the use of additional radiotherapy, but also the use of SLNB. Ongoing clinical trials aiming to identify a subgroup of low risk DCIS also base identification of this subgroup on histologic grade [40, 41]. DCIS grading is based on morphologic characteristics, such as growth pattern, cytoplasmatic feature, nuclear pleomorphism and mitotic activity. Since diagnostic criteria are not always clear, differences in morphological interpretation do make the accuracy of DCIS grading questionable [42, 43]. Consequently, histologic grading of DCIS is currently not meeting high enough standards [44]. Improvement of the accuracy is extremely relevant, since grade is the most important determinant for the management of DCIS at the moment. Recent studies on molecular alteration driving the progression of DCIS towards invasive breast cancer, show that gene expression profiling can possibly improve the ability to predict progression to invasive breast cancer [45–47]. This suggests that more effective methods of detecting, diagnosing and treating DCIS can be developed based on targeting these genes, resulting in more individualized treatments in the near future. However, gene expression profiling is still very expensive and recent studies suggest that the use of a free-of-charge online Nomogram (available online at www.nomograms.org) is concordant with those obtained using the commercially available DCIS scores for women aged 50 years or older with small DCIS (≤ 2.5 cm) [48].

This study has several limitations. The study population, selected from the NCR, was not manually controlled using the PALGA database. A previous study by Elshof et al., also using data from the NCR, has shown that not all DCIS patients in the NCR database consisted of pure DCIS when checked in the PALGA database [14]. Therefore, our results on the iRFS must be interpreted considering this misclassification, especially in the older years, which may have caused a too low iRFS rate. Furthermore, the follow-up in our iRFS analysis for recent years (2011–2018) is still short. In addition, the iRFS analysis only contains invasive relapses. Data on non-invasive relapse were not available. Data on overall survival were not included in this study, since it has already been described that DCIS patients have a higher risk of dying from breast cancer compared with the general female population, but absolute ten-year risks are very low [49].

In conclusion, the use of BCS, radiotherapy and axillary staging in patients with DCIS varies over time. The incidence of BCS increased over the years with a decline in the use of adjuvant radiotherapy and SLNB, especially for low grade DCIS, in more recent years. The lack of consensus in recent literature reflects our limited knowledge about the natural progression of untreated DCIS. Because of this dilemma, current treatment protocols may be too defensive and result in overtreatment of many women. Therefore, more research is needed to help prevent overdiagnosis and overtreatment in the future.

## Data Availability

Available upon request.

## Code availability

SPSS, version 24.0 (SPSS, Inc., Chicago, USA).
